# Human Adipose-Derived Mesenchymal Stem Cells Cryopreservation and Thawing Decrease *α*4-Integrin Expression

**DOI:** 10.1155/2016/2562718

**Published:** 2016-02-15

**Authors:** Ana Carolina Irioda, Rafael Cassilha, Larissa Zocche, Julio Cesar Francisco, Ricardo Correa Cunha, Priscila Elias Ferreira, Luiz Cesar Guarita-Souza, Reginaldo Justino Ferreira, Bassam Felipe Mogharbel, Venkata Naga Srikanth Garikipati, Daiany Souza, Mirian Perlingeiro Beltrame, Katherine Athayde Teixeira de Carvalho

**Affiliations:** ^1^Cell Therapy and Biotechnology in Regenerative Medicine Research Group, Pelé Pequeno Príncipe Institute, Avenida Silva Jardim 1632, 80250-200 Curitiba, PR, Brazil; ^2^Bioprocess Engineering and Biotechnology Department, Federal University of Paraná, Avenida Coronel Francisco Heráclito dos Santos 210, 81531-970 Curitiba, PR, Brazil; ^3^Hospital Santé Curitiba, Plastic Surgery Clinic, Rua XV de Novembro 2913, 80045-340 Curitiba, PR, Brazil; ^4^Experimental Laboratory of Institute of Biological and Health Sciences of Pontifical Catholic University of Paraná (PUCPR), Rua Imaculada Conceição 1155, 80215-901 Curitiba, PR, Brazil; ^5^Center for Translational Medicine, Temple University School of Medicine, Philadelphia, PA 19140, USA; ^6^Flow Cytometric Analysis Laboratory, Clinical Hospital of Federal University of Paraná, Rua Padre Camargo 280, Alto da Glória, 80060-240 Curitiba, PR, Brazil

## Abstract

*Aim*. The effects of cryopreservation on adipose tissue-derived mesenchymal stem cells are not clearly documented, as there is a growing body of evidence about the importance of adipose-derived mesenchymal stem cells for regenerative therapies. The aim of this study was to analyze human adipose tissue-derived mesenchymal stem cells phenotypic expression (CD34, CD45, CD73, CD90, CD105, and CD49d), colony forming unit ability, viability, and differentiation potential before and after cryopreservation.* Materials and Methods*. 12 samples of the adipose tissue were collected from a healthy donor using the liposuction technique. The cell isolation was performed by enzymatic digestion and then the cells were cultured up to passage 2. Before and after cryopreservation the immunophenotype, cellular viability analysis by flow cytometer, colony forming units ability, differentiation potential into adipocytes and osteoblasts as demonstrated by Oil Red O and Alizarin Red staining, respectively.* Results*. The immunophenotypic markers expression was largely preserved, and their multipotency was maintained. However, after cryopreservation, the cells decreased *α*4-integrin expression (CD49d), cell viability, and number of colony forming units.* Conclusions*. These findings suggest that ADMSC transplanted after cryopreservation might compromise the retention of transplanted cells in the host tissue. Therefore, further studies are warranted to standardize protocols related to cryopreservation to attain full benefits of stem cell therapy.

## 1. Introduction

In the development of new therapies using stem cells, mesenchymal stem cells (MSC) from diverse origins have exhibited immense potential [1, 2]. Although the reports related to the therapeutic potential of MSC, most of them are performed mainly on hematopoietic origin, more from bone marrow (BM), that is explained by the extensive clinical experiments with stem cells, particularly in the treatment of oncohematological diseases [3, 4]. However, adipose tissue-derived mesenchymal stem cells (ADMSC) emerged as a promising candidate. As ADMSC are adult stem cells that can be isolated easily from adipose tissue, the cells are obtained through plastic surgery that represents an ample and accessible source of adult stem cells with the ability to differentiate into adipocytes, osteocytes, chondrocytes, and cells phenotypically similar to neurons [5]. Due to ADMSC ability to differentiate into several cell types of clinical interest, they hold an immense potential for the future therapeutic use of these cells in clinics. Although many reports related to the therapeutic potential of ADMSC, most of the current investigations were performed mainly on freshly isolated cells [6]. As this cell type holds a clinical translation value, there is an increasing interest in stem cell banking of ADMSC.

In consequence of the inherent characteristics of the ADMSC: low immunogenicity, higher cellular yield, in comparison to bone marrow cells and pluripotency, the cryopreservation of these cells would be a very convenient alternative. However, the effects of the cryopreservation and the thawing proceedings could impact their therapeutic outcome and therefore should be evaluated. Once the cells are cultivated and are attached to the plastic, before the cells cryopreservation, these are subjected to a subtract desegregation stress, being able to put in risk the integrity of the cell membrane and its surface molecules for the cell adhesion. After thawing, the cryopreserved cells have also the risk of injuring the membrane and other cellular functional characteristics; both proceedings could affect the therapeutic outcomes.

In this context, the present study aims to address whether the ADMSC cryopreservation could compromise the cell integrity, the expression of adhesion molecules, and/or the differentiation potential of ADMSC and other stem cells lineages.

## 2. Material and Methods

### 2.1. Experimental Design

Adipose-derived mesenchymal stem cells were obtained from adipose tissues of 12 adult healthy donors (10 female and two male) by liposuction performed by a plastic surgeon with informed consent signature from each patient. The cell isolation was performed by enzymatic digestion with collagenase type I. Then, the cells were cultured in DMEN/F12 supplemented with 10% of calf fetal serum, 100 units/mL of penicillin, and 100 *μ*g/mL of streptomycin. Before and after cryopreservation, the following assays were performed: colony forming units, immunophenotype, and cellular viability with Annexin V and 7-AAD analysis by flow cytometer as well as ADMSC differentiation to adipocytes and osteoblasts.

### 2.2. Isolation of ADMSC

The adipose tissue samples were collected from a healthy donor after consent signature from each patient. All the samples were collected from specialized plastic clinics, using the liposuction technique. After the samples were collected, they were cultured before 24 hours, using the following protocol: 50 mL of each sample was extensively washed with phosphate buffered saline (PBS) (Sigma, St. Louis, MO, USA) containing 300 units/mL of penicillin and 300 *μ*g/mL of streptomycin (Sigma, St. Louis, MO, USA) (PBS/PS). After washing the samples and debris removal, these were placed in 50 mL tubes with 0.075% of type I A collagenase (Sigma, St. Louis, MO, USA), prepared in PBS/PS. The cells were homogenized and incubated with agitation at 37°C for 30 minutes. After the incubation, the type I collagenase activity was ceased by adding the same volume of Eagle modified from Dulbecco/F12 (DMEM/F12) (LGC Biotechnology, Brazil), containing 10% of fetal calf serum (FCS) (GIBCO B Life Technologies, Inc., Rockville, USA, RL) as determined to be standard culture medium (SCM). Then, the samples were centrifuged at 400 g for 10 minutes. The supernatant was discarded and the pellet was resuspended in 10 mL of PBS/PS; then, another centrifugation was made at 400 g for 10 minutes. The supernatant was discarded and the pellet was suspended in 5 mL of culture medium. The cell suspension was filtered in cell strainer of 100 *μ*M (Becton Dickinson, USA). The cell count was made by a hemocytometer and the utilization of Trypan Blue (Sigma, St. Louis, MO, USA) (for the dilution of 1 : 1, 10 *μ*L of the cell suspension was added for 10 *μ*L of Trypan Blue). After that, 1 × 10^5^ cells for cm^2^ in 25 cm^2^ and 75 cm^2^ culture flasks were harvested and incubated at 37°C and 5% of CO_2_ [7–9].

### 2.3. Cultivation and Expansion

72 hours after, on cultivation, the medium was aspirated from the flasks and the cells were washed with preheated PBS/PS; then, 7 mL of culture medium was added in the 25 cm^2^ and 12 mL in the 75 cm^2^ flasks. The cells were kept incubated at 37°C and 5% CO_2_. The medium was changed twice a week until the cells reached between 80% and 90% of confluence. After the desired confluence was reached, the cells were briefly washed with preheated PBS/PS and added trypsin/EDTA (0,25%) (Sigma, St. Louis, MO, USA) for disaggregation of the adherent cells. The cells were incubated for 10 minutes at 37°C. Then, the same volume of SCM was added for the neutralization of the trypsin action. The cell suspension was transferred to sterile centrifuge tube and centrifuged at 400 g for 10 minutes. The supernatant was discarded and the cells were resuspended in approximately 5 mL of SCM. The cell count was realized with a hemocytometer chamber, using Trypan Blue (1 : 1 dilution) [9]. After the counting, cells were once again harvested in 75 cm^2^ flasks at a concentration of 1 × 10^3^ cells/cm^2^ and incubated at 37°C and 5% of CO_2_ until they reached 80% of confluence. When they reached the desired confluence, the cells were trypsinized and were available for analysis. For each sample, the culture was done at passage 2 (P2).

### 2.4. Cryopreservation Proceedings

After trypsinization at P2 cells as described previously, and confirming their viability by the Trypan Blue exclusion method, a fraction of the cells were submitted to the cryopreservation proceedings. The cells were counted with a hemocytometer (approximately, 1 × 10^6^ cells/mL). The cryoprotectant medium had 80% of BFS, 10% of dimethyl sulfoxide (DMSO), (Sigma, St. Louis, MO, USA), 10% of DMEM-F12 (LGC Biotechnology, Brazil), and 100 units/mL of penicillin and 100 *μ*g/mL of streptomycin (Sigma, St. Louis, MO, USA). 1 mL aliquots (containing 1 × 10^6^ cells) were transferred to preidentified cryogenics tubes (cell type, density, and date). The tubes were cooled in a programmable freezing device (Nicool LM10; Air Liquide, Marne La Vallée, France). The cryopreservation equipment starts at room temperature and in the end of the first stage (Program 3–15 minutes) reaches the temperature −30°C; in the end of step 2 (Program 5–45 minutes), the temperature is −60°C; finally, in the last stage, step 3 (Program 9-10 minutes), the temperature is −110°C. After step 3, the cells were transferred to the liquid nitrogen to −196°C. This method prevents the crystals formation inside the cell, because the freezing occurs slowly [9–11]. Shortly after, the tubes were transferred to the liquid nitrogen where they remained stocked for approximately 20 days. All the samples were cryopreserved in triplicate.

### 2.5. Thawing Proceedings

20 days after, the cryopreserved cells were thawed at 37°C and transferred in 5 mL of SCM and gently homogenized. The cell suspension was transferred for sterile centrifuge tube and centrifuged at 400 g for 10 minutes. The supernatant was discarded and the cells were resuspended in approximately 5 mL of culture medium. The cells were submitted to analysis.

### 2.6. Colony Forming Units (CFU) Analysis

Before and after the cryopreservation, 10 cells per cm^2^ were seeded, in triplicate; in wells of 9 cm^2^ with SCM, the medium was changed according to other steps of culture. After 14 days, the medium was removed and the cells were fixated with methanol for 5 minutes and then stained with 0.5% of crystal violet in methanol for more 5 minutes. The flasks were washed twice with PBS/PS and dried. The number of colonies with more than 2 mm was counted and the results represent the number of former colonies by 100 seeded cells (counted colonies/inoculated cells) × 100 [12].

### 2.7. Immunophenotypic Analysis

All samples were analyzed for the expression of surface markers (Table 1), using monoclonal antibodies against cluster of differentiation (CD) antigens, conjugated with fluorochromes and analyzed, before the cryopreservation and after thawing, by flow cytometer. Cells (number) were incubated with the antibodies that are described in Table 1 and corresponding isotypes were used. The primary antibodies were incubated for 10 minutes in the dark. The antibody against CD105 is a purified antibody and it is not conjugated with fluorescence; for this reason, it was necessary to incubate it with a secondary antibody for more 15 minutes in the dark room and redo the cell washing step. Further 5 *μ*L of 7-AAD was added and incubated in the dark room for 15 minutes. After that, 400 *μ*L of binding buffer was added and the samples were analyzed by flow cytometer (FACS Calibur; Becton Dickinson, San Diego, USA) [11, 13, 14]. Prior to each test, the equipment was calibrated using BD Calibrite (Becton Dickinson, San Diego, USA), according to the manufacturer's instructions. All antibodies were used according to the manufacturer's instructions. 20.000 events (cells) were captured; the percentage values of each marker were analyzed through specific analysis software: Cyflogic version 1.2.1.

### 2.8. Cell Viability with Annexin V and 7-AAD Analysis

The adherent cells were detached with trypsin (0.25%), centrifuged for 3 minutes at 400 g, and resuspended in 2 mL of PBS. After that, in 5 mL tubes, 100 *μ*L of this solution was added. After that, 400 *μ*L of PBS was added in each tube; they were homogenized and centrifuged. The supernatant was discarded and 100 *μ*L of the binding buffer was added. Afterwards, 5 *μ*L of Annexin V was added, 5 *μ*L of 7-AAD was added, and the tubes were incubated in the dark room for 15 minutes. After incubation, 400 *μ*L of binding buffer was added and homogenized; the data was collected by flow cytometer (FACS Calibur; Becton Dickinson, USA) [11].

### 2.9. Adipogenic Differentiation

The cells were treated with adipogenic medium consisting of DMEM-F12 medium containing 10% FBS, 0.5 m M isobutylmethylxanthine (Sigma, St. Louis, MO, USA), 1 mmol/L dexamethasone (Sigma, St. Louis, MO, USA), 10 mg/mL insulin, and 50 *μ*M indomethacin (Sigma, St. Louis, MO, USA) and were incubated at 37°C and 5% CO_2_. Control cells were treated with SCM. Medium was changed two times a week. 14 days after, the cells were fixed and stained with Oil Red Stain (Sigma, St. Louis, MO, USA) to demonstrate the fat droplets in the cells [7, 8].

### 2.10. Osteogenic Differentiation

The osteogenesis was induced by keeping the cells in DMEM-F12 medium with 100 units/mL of penicillin, 100 *μ*g/mL of streptomycin, 1 nM of dexamethasone, 2 mM of *β*-glycerophosphate (Sigma, USA), and 50 *μ*M of ascorbate-2-phosphate (Sigma, USA) and incubated at 37°C and 5% CO_2_. Control cells were treated with SCM. The cells were kept in these conditions for 35 days and the medium was changed twice per week. The experimental and control cells were fixed glutaraldehyde at 2.5% for ninety minutes and stained with Alizarin Red Stain (Sigma, St. Louis, MO, USA) to demonstrate mineralization [7, 8].

### 2.11. Statistic Analysis

The obtained results in this study were expressed as mean ± standard deviation (SD). For the comparison between the molecules expression analysis before and after cryopreservation, the *t*-test was performed; *p* < 0.05 was considered significant. The data were organized in Excel spreadsheet and were analyzed with Statistica version 9.0 software.

## 3. Results

### 3.1. Phenotypic Analysis of ADMSC

**Figure 1:**
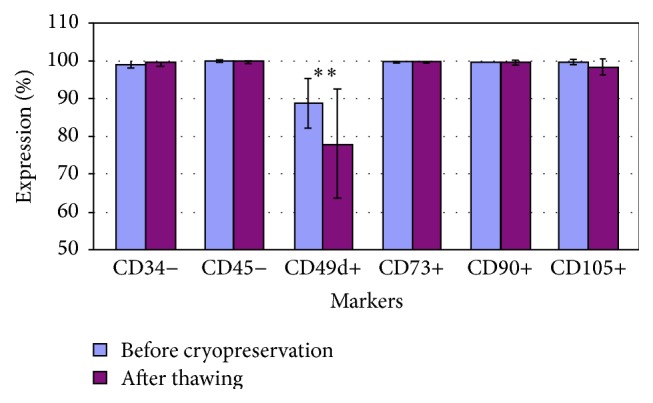
Surface markers expression of cells before cryopreservation and after thawing. The analysis was done by Cyflogic software 1.2.1. The *t*-test was done; *p* < 0.05 was considered significant. The results were *p* ^*∗∗*^ = 0,001.

**Figure 2:**
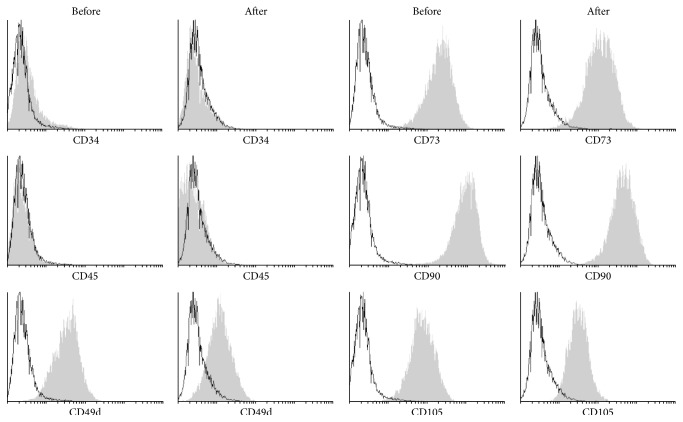
Histograms of ADMSC markers before and after cryopreservation. The grey color represents specific marker and the white color represents an isotype control.

Flow cytometric analysis showed a typical mesenchymal phenotype of ADMSC with expression of CD73, CD90, and CD105 whereas these cells lacked expression of CD34 and CD45. Interestingly, we observed a significant reduction of CD49d expression after thawing cryopreserved ADMSC (Table 2; Figures 1 and 2).

### 3.2. Annexin V 7-AAD Staining

**Figure 3:**
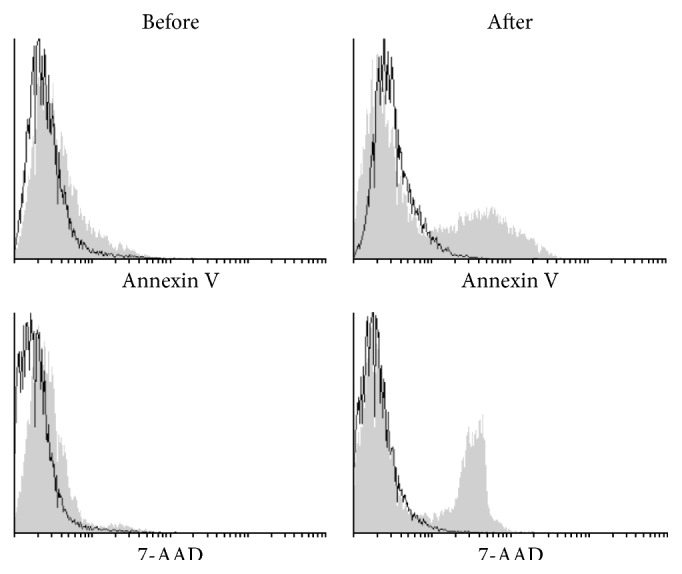
Histograms of Annexin V (apoptosis marker) and 7-AAD (viability marker) of the cells before and after cryopreservation. The grey color represents specific marker and the white color represents an isotype control.

The differences in CD49d expression before and after cryopreservation led us to look at the cell viability before and after cryopreservation. Cell viability was assessed by Annexin V 7-AAD staining; we observed a significant reduction in viability from 91.34%  ±  4.54% to 74.99%  ±  14.19% (*p* = 0.001) after cryopreservation, losing an average of 17.9% viable cells. Concerning labeling with Annexin V (apoptosis), values were very close to the values of cellular viability, being 91.39%  ±  5.5% before cryopreservation and 76.31% ± 13.33% after thawing (*p* = 0.003) (Table 3; Figure 3). Thus, suggesting that, the majority of Annexin V stained cells were also stained with 7-AAD, which means that the amount of cells only in apoptosis was a small proportion.

### 3.3. Colony Formation Assay

Further, we looked at the colony formation ability of ADMSC and observed a significant decrease in the colonies formation capacity; CFUs before and after cryopreservation were 28.08%  ±  7.06% versus 21.51%  ±  6.61% (*p* < 0.01).

### 3.4. Adipogenic Potential of ADMSC

**Figure 4:**
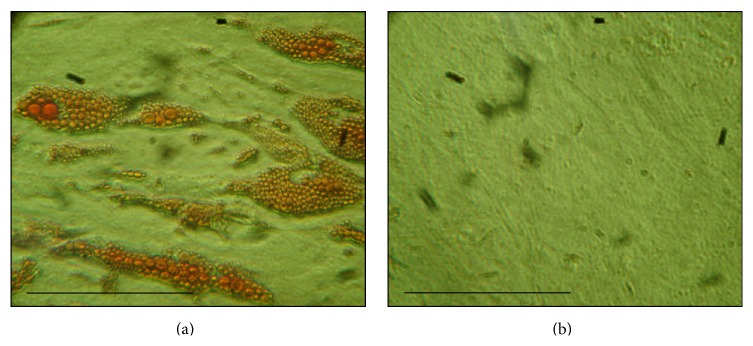
Adipose differentiated cells after 14 days in induction medium: sample after thawing of cryopreserved cells, phase contrast microscopy, 250x. (a) Presence of fat droplets (stained with Oil Red) in ADMSC cultivated with adipogenic induction medium. (b) Control does not have fat droplets, indicating the undifferentiated cells cultivated with standard medium. Scale (10 *μ*m).

It was assessed, after cryopreservation with a lineage-specific induction medium, the cells differentiated into adipogenic as evidenced by Oil Red, whereas control cells did not take up Oil Red Staining (Figure 4).

### 3.5. Osteogenic Potential of ADMSC

**Figure 5:**
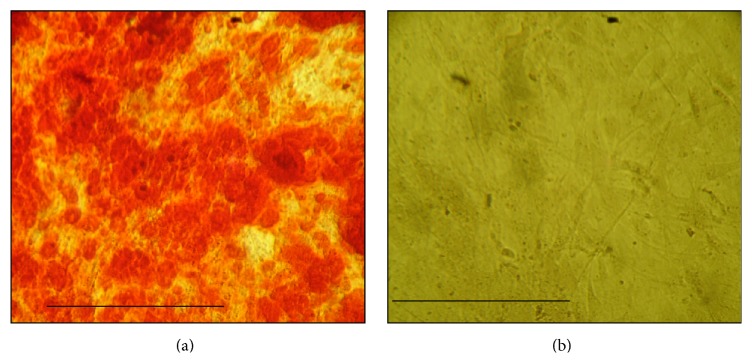
Osteogenic differentiated cells after 35 days in osteogenic induction medium: sample after thawing of cryopreserved cells, 250x rise. (a) ADMSC stained with Alizarin Red, demonstrating the presence of calcium in extracellular matrix. (b) Control without matrix demonstration, indicating the undifferentiated cells cultivated with standard culture medium. Scale (10 *μ*m).

In addition, upon treatment with a lineage-specific induction medium, the cells differentiated into osteogenic as evidenced by Alizarin Red, whereas control cells did not take up Alizarin Red staining (Figure 5).

## 4. Discussion

The ADMSC are cells with a huge therapeutic potential and important tool to cellular metabolism studies. Therefore, the characterization of cryopreserved cells and their maintenance after thawing cryopreserved are relevant.

About the CFU analysis, we observed a significant decrease in the colonies formation capacity; CFUs before and after cryopreservation were 28.08%  ±  7.06% versus 21.51%  ±  6.61% (*p* = 0.01), respectively. These results are in agreement with the results found by Goh and colleagues (2007) that cryopreservation causes decrease in adhesion efficiency of ADMSC [15]. This difference could be related to decreased expression of integrin *α*4 (CD49d) that we have observed. Mitchell et al. (2006) reported that the ability to form colonies of ADMSC is considerably greater than BMSC, even after cryopreservation. This group also reported that the more the passages, the greater the number of CFUs and suggests that the passages select a particular cell type, possibly the stem cells [16]. The same parameters were kept before and after cryopreservation and, however, can indeed be a delay in cell growth, considering the fact that the cells were again adapting to cultivation conditions and the limited size of the colonies. Regarding cellular therapy, the increase of number of the passages could compromise the safety and that is why it may be related to increasing mutations and increasing the transformation of the cells [17]. On the other hand, about the differentiation process, most of the groups studying with ADMSC report that, after 14 days of adipogenic induction, all samples of cells have a morphology consistent with adipocyte, which was confirmed by staining of lipids with Oil Red as well as by PCR that identify genes consistent with adipocytes and which are not present in undifferentiated cells. Rodriguez and colleagues (2004) reported that after this period about 90% of the cells accumulate intracellular lipid and that there might also be no significant accumulation in untreated cells [18]. All samples, before and after thawing, that were induced to the adipogenic differentiation medium stained positive for Oil Red, showing the presence of lipids inside the cells (Figure 4(a)). In the control medium, the standard culture medium was added and there was no trace of lipids, indicating that these cells did not differentiate into adipocytes (Figure 4(b)). These results clearly show that ADMSC have after appropriate inducing an adipogenic differentiation capacity. To identify osteogenic differentiation, several groups have reported that, between 14 and 21 days after induction, we can observe the presence of a mineralized matrix, by the calcium phosphate staining with Alizarin Red [16, 19]. In the present work, it was observed only after 30 days in the induction medium. All samples, before and after thawing, that were induced to the osteogenic differentiation medium stained positive for Alizarin Red (Figure 5(a)). In the control medium, the standard culture medium was added and there was no evidence of mineralization, indicating that these cells did not differentiate into osteogenic differentiation (Figure 5(b)). Both adipogenic and osteogenic differentiation results suggest that cells were stem cells. Other researchers reported the maintenance of the capacity of differentiation of ADMSC in both adipocytes and osteocytes after thawing [19–21]. In this study, 100% of the samples had the same induction time.

There are some controversies about the markers for MSC. This occurs because of different designs of different types of markers; however, there is a consensus that MSC are negative for CD45 (a marker of HSC) [22]. In this study, all samples were negative for CD45, which is an indication that they would be large MSC. In this study, four samples showed a small population of cells positive for CD34 (1 to 3%).

In this study, varying values for the CD49d were found (88.67 ± 6.55), consistent with the values found by Katz and colleagues (2005) (78 ± 20) [23]. Maybe, if those cells will be submitting culture again, it will be possible to recover this expression of CD49d. In this work, cultivating the cells after cryopreservation was not possible due to the low number of cells. However, thinking about translation to humans, the most likely hypothesis in a cell therapy would be the immediate transplant of the cells after thawing. Thus, as already described in the literature, increasing the number of passages also increases the probability of cell transformations such teratomes, which makes impracticable therapy [18]. In addition to varying values of expression, this surface marker showed significant decreases after thawing of cryopreserved cells (77.8 ± 14.45, *p* = 0.007). This marker represents the *α*4-integrin, an adhesion molecule that interacts with the *β*1 integrin forming a heterodimer, late activating antigen-4 (VLA-4) [13]. Lei and colleagues (2007) found low positive values for CD49d (average 12.6%), but the way of isolation of ADMSC was through the subcutaneous tissue and not through lipoaspirate; another issue is that they used DMEM with low glucose; these differences protocols can select different types of cells with similar characteristics, but not identical [24]. For Katz and coworkers (2005), some differences between groups with respect to expression of CD49d reflect adjustments of countless variations of these cells to the extracellular medium, such as density, cell cycle, culture time, and the number of passages [23]. The environment produces appropriate niches for stem cells and regulates the maintenance of these niches for specific cell lines. For this regulation, the adhesion of stem cells in the extracellular matrix is critical as it allows communication between the cells and the matrix, being a prerequisite for the maintenance of tissue [25]. Thus, these changes in expression of CD49d after cryopreservation may mean a major problem in transplantation of these cells, causing them not properly communicating with the injured tissue.

In this study, only viable and intact cells were analyzed. Gonda et al. (2008) stated that cryopreservation can cause structural and functional damage to cell proteins and reduce their viability, but immunophenotypic exchanges could hardly occur [19]. Expression changes really would not have excuses, but the loss of expression is very relevant because the group itself mentioned that cryopreservation can cause damage to the membrane protein, which is the case of CD49d, which represents an adhesion protein. Few studies are related to immunophenotypic difference after thawing of cryopreserved cells, which is an extremely important feature that should be studied [19, 26]. Cellular viabilities before cryopreservation and after thawing were analyzed by staining with the kit of viability (BD) Annexin V PE_7-AAD on 20,000 events. Apoptosis of the cells is characterized by phosphatidylserine, a component of the inner leaflet of cell membranes. When a cell enters apoptosis process, phosphatidylserine becomes exposed on the outer wall of the membrane, but the cell membrane remains intact. The cells positive for Annexin V represent cells that have this translocation of phosphatidylserine [27]. After thawing there was a significant loss of the cells integrity, being 16.5% lower than the cells before the cryopreservation (Figure 5). Maybe that cell integrity is also lost with thawing? Another cells line, which was positive for 7-AAD, indicates that the membrane integrity was compromised; consequently, there was cell death. Accordingly, these markers are used to distinguish dead cells from cells that are in the process of apoptosis. To analyze these markers, Cyflogic software 1.2.1 was used, which provides the histogram overlay of the isotype control with the results of fluorescence of each marker and quantifies the percentage of positive (not overlapping the isotype control) and negative (superimposed on the isotype control) markings. Thus, it was possible to analyze three variables: (i) 100% viable cells (negative for Annexin V-PE and negative for 7-AAD); (ii) dead cells (positive for Annexin V-PE and positive for 7-AAD); and (iii) the process of apoptosis in cells (positive for Annexin V-PE and negative for 7-AAD). Following these parameters, before cryopreservation, viability was 91.34%  ±  4.54%. After thawing, the cells had a significant drop in cell viability, 74.99%  ±  14.19% (*p* = 0.001), losing on average 17.9% viable cells. Concerning labeling with Annexin V (apoptosis), values were very close to the values of cellular viability, being 91.39%  ±  5.5% before cryopreservation and 76.3% ± 13.33% after thawing (*p* = 0.003) (Table 3). This study demonstrates that the majority of Annexin V stained cells were also stained with 7-AAD, which means that the amount of cell only in apoptosis was small.

The ADMSC viabilities of cryopreserved cells after thawing may be explained with the concentration of cells in each cryotube. Goh et al. (2007) tested four cell concentrations: 2.5 × 10^5^, 5 × 10^5^, 1 × 10^6^, and 2 × 10^6^ per mL and found a viability of 71.4%, 81.10%, 77.9%, and 69.2%, respectively. In this study, the cryopreservation of cells in 1 × 10^6^ cells per mL and viability found values similar to values found by Goh group (2007); however, the method used by Goh et al. (2007) was staining by Trypan Blue which is more relative to be counted manually; the method used in this study is more accurate, by flow cytometric analysis [15]. Thirumala and colleagues (2010) found viabilities, staying at 84%  ±  8% when using the same cryoprotectant in their study, but the test was performed on P1 [27]. De Rose and colleagues (2009) found amazing values of cellular viability 92.5%. This high rate of viability may be related to the form of thawing these cells, which were transferred to culture medium with 10% FCS prior to complete thawing; it could be explained by the fact that the cells stayed less time in contact with DMSO in room temperature that is known for its cytotoxic effects [10]. The researchers have shown that many factors influence the intracellular dynamics when cells are frozen, affecting the viability of these cells. Among these factors can be highlighted the formation of intracellular ice, which can perforate the cell membranes, and high concentration of cells which can limit the space preventing cell growth during freezing [15]. A standard protocol was used for various cell types, but some cells have specific characteristics, which may require special care. For this reason, it would be ideal to develop a specific protocol for ADMSC, to improve the viability rate and other characteristics [11, 19, 28].

Some authors used as long-term storage method freezing in a freezer at −80°C rather than liquid nitrogen (−196°C), but this method has revealed lower levels of viability while retaining the functional characteristics. Other variables could influence the viability, like the speed of freezing, because if it will be faster, there are greater probabilities of intracellular ice formation and consequent membrane damage [19, 26] and the choice of serum free media, which may not benefit from a large cell viability. However, considering therapeutic applications, even with this, low viability could be indicated by the reduced risk of contaminating [26].

## 5. Conclusions

In this study, the standard protocols of the human adipose-derived mesenchymal stem cells cryopreservation and thawing proceedings are demonstrating the decrease: *α*4-integrin expression (CD49d), cell viability, and colony forming units after thawing. These findings can compromise the integration of cells in the extracellular matrix of the host tissue. Further protocols should be established to improve and ensure the cell graft.

## Figures and Tables

**Table 1:** Panel of monoclonal antibodies used in this study for immunophenotypic characterization.

Marker	Clone	Reactivity	Fluorochrome	Function
CD34	581/CD34	Human	FITC	Stem cell marker (precursor), also found in hematopoietic progenitor cells, vascular endothelium, and fibroblasts of the same tissue. Probably, it works as a transduction signer and has a function in endothelium specific antigen adhesion.

CD45 (common leukocyte antigen)	HI30	Human	PE	Leukocytes marker. CD45 proteins are located in all hematopoietic cells, but not in erythrocytes.

CD49d (integrin *α*4)	9F10	Human	PE	Transmembrane glycoprotein, integrin *α*4. It makes several cell-cell interaction and cell-matrix and participated in cellular adhesion.

CD73 (ecto-5′-nucleotidase)	AD2	Human	PE	It have been suggested that this marker can mediate costimulator signals in the cells T activation and endothelium as well catalyses the dephosphorylation of adenosine monophosphate in adenosine.

CD90 (THy-1)	5E10	Human	FITC	The role of interaction in cell-cell and cell-matrix has been speculated, with related neurites growth, nerve regeneration, apoptosis, metaphase, inflammation, and fibrosis.

CD105 (endoglin)	266	Human	Purified	Responsiveness modulator of TGF-*β* cellular complex.

**Table 2:** Surface markers expressions before cryopreservation and after thawing.

Molecular marker	CD34−		CD34+		CD45−		CD49d+		CD73+		CD90+		CD105+	
Cryopreservation	Before	After	Before	After	Before	After	Before	After	Before	After	Before	After	Before	After
Medium	98.88	99.3	1.12	0.78	99.79	99.8	88.67	77.8	99.57	99.5	99.55	99.5	99.4	98.3
Max	99.86	100	3.32	2.1	100	100	96.38	93.1	99.94	99.9	99.97	100	99.91	99.9
Min	96.98	97.9	0.14	0.03	99.27	99.6	80.15	41.6	98.51	98.5	98.15	97.5	97.87	93.9
SD	1.08	0.61	1.08	0.6	0.22	0.14	6.55	14.5	0.43	0.43	0.07	0.7	0.64	2.07

*p* value	**0.113**		**0.158**		**0.791**		**0.007** ^**∗**^		**0.528**		**0.618**		**0.05**	

^*∗*^ *p* < 0.05.

**Table 3:** Representation of viability and integrity cells before cryopreservation and after thawing.

	Annexin V		7-AAD	
	Before	After	Before	After
Media	91.39	76.31	91.34	74.99
Max	96.2	95.83	79.29	49.21
Min	75.18	52.38	95.17	95.27
DP	5.85	13.33	4.54	14.19

*p* value	**0.003**		**0.001**	
